# The DYF-5 RCK and CDKL-1 CDKL5 kinases contribute differentially to shape distinct sensory neuron cilia morphologies in *C. elegans*

**DOI:** 10.17912/micropub.biology.000619

**Published:** 2022-08-04

**Authors:** Ashish Kumar Maurya, Piali Sengupta

**Affiliations:** 1 Department of Biology, Brandeis University, Waltham, MA, USA

## Abstract

The conserved CCRK, RCK, and CDKL5 kinases regulate cilia length in diverse organisms. In *C. elegans* , DYF-18 CCRK regulates DYF-5 RCK to shape both simple and complex cilia morphologies. The CDKL5 ortholog CDKL-1 has also been suggested to act downstream of DYF-18 but independently of DYF-5 to regulate lengths of simple rod-like cilia. Here we show that CDKL-1 is largely dispensable for regulation of complex cilia structures. Using genetic epistasis experiments, we confirm that CDKL-1 and DYF-5 act independently to control cilia architecture. Our results indicate that multiple kinases act via distinct pathways to regulate unique cilia ultrastructures.

**Figure 1. CDKL-1 acts independently of DYF-5 to regulate morphology of simple but not complex cilia in C. elegans .**
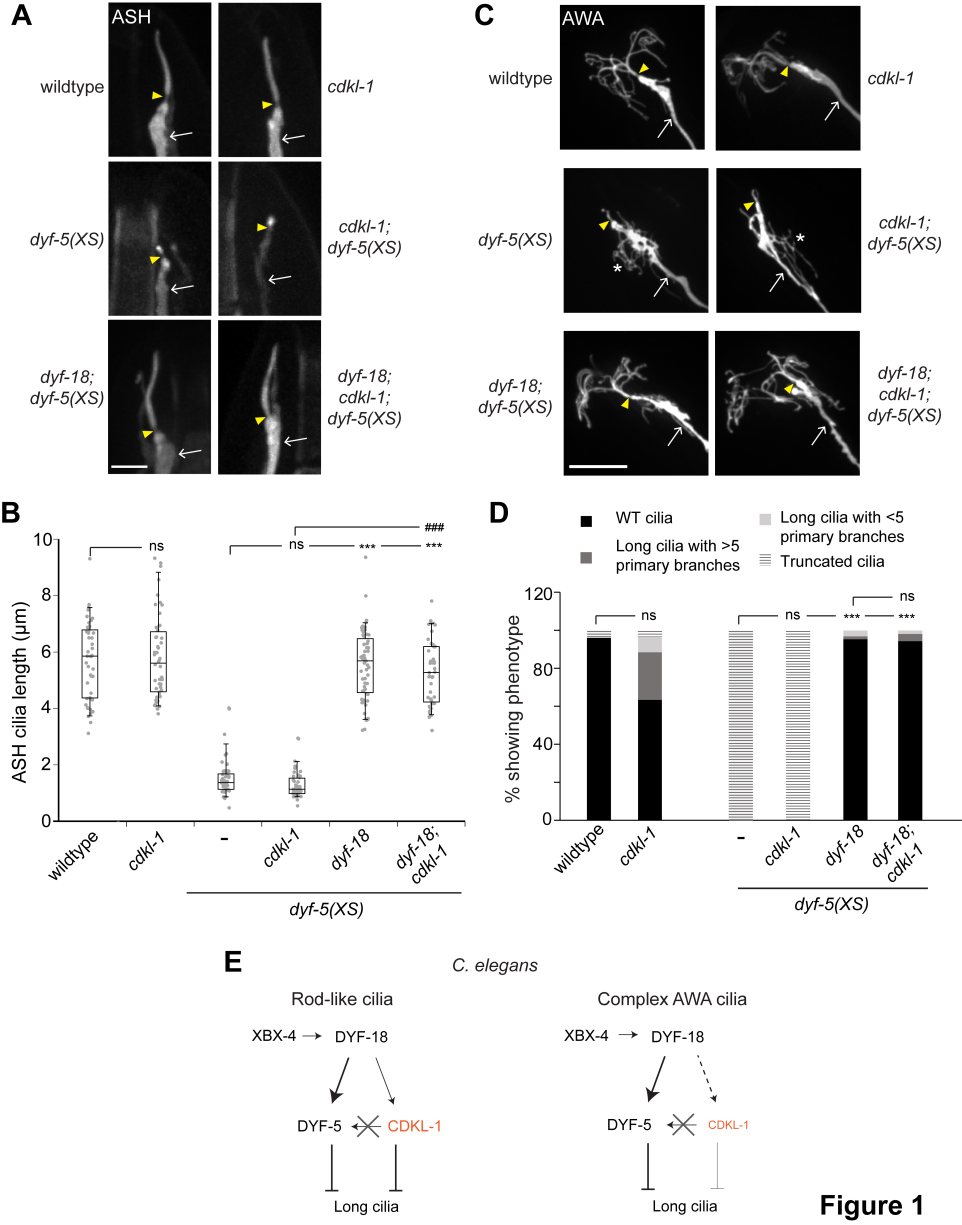
**A,B)** Representative images of ASH cilia (A) and quantification of ASH cilia lengths (B) in the indicated genetic backgrounds. ASH cilia were visualized via expression of *sra-6* p *::myr-gfp* . Arrowheads indicate cilia base; arrows indicate dendrite. Anterior is at top. Scale bar: 4 μm. **C,D)** Representative images of AWA cilia (C) and quantification of AWA cilia morphologies (D) in the indicated genetic backgrounds. AWA cilia were visualized via expression of *gpa-4pΔ6* p *::myr-gfp* . Arrowheads indicate cilia base; arrows indicate dendrite; asterisk indicates dendritic branches. Anterior is at top left. Scale bar: 10 μm. Alleles used were: *cdkl-1(ok2694)* and *dyf-18(ok200)* . Images shown are from adult hermaphrodites. *** and ###: different between indicated at *P* < 0.001 (one way ANOVA with Bonferroni posthoc test (B), Kruskal-Wallis with Bonferroni posthoc test (D). ns – not significant. n ≥30 neurons each. **E)** In neurons containing rod-like cilia, DYF-18 acts upstream of both DYF-5 and CDKL-1; DYF-5 and CDKL-1 act independently to inhibit cilia length. In the AWA neurons, DYF-18 also acts upstream of DYF-5 to regulate their complex cilia architecture. CDKL-1 appears to play only a minor role in shaping AWA cilia. XBX-4 regulates DYF-18 in multiple sensory neuron types (Maurya and Sengupta, 2021).

## Description

Cilia and flagella are compartmentalized microtubule-based structures that mediate cell motility and/or sensory functions (Malicki and Johnson, 2017; Spassky and Meunier, 2017). These organelles are typically rod-like structures although a subset of cilia present on sensory neurons exhibit diverse and complex morphologies (Silverman and Leroux, 2009). Cilia length and architecture are strictly regulated as a function of cell type and conditions across organisms and play important roles in tuning cellular responses (e.g. Lattao et al., 2017; Menco, 1997; Pan and Snell, 2000; Perkins et al., 1986; Rosenbaum et al., 1969). The genetic pathways that modulate cilium structures remain to be fully characterized.

The highly conserved CCRK and RCK kinases have been shown to act in a cascade to restrict cilia length in organisms ranging from the blue-green algae *Chlamydomonas reinhardtii* to mammals (Asleson and Lefebvre, 1998; Berman et al., 2003; Broekhuis et al., 2014; Burghoorn et al., 2007; Jiang et al., 2019; Maurya et al., 2019; Moon et al., 2014; Tam et al., 2007). Loss of function of these kinases elongates cilia in part via modulation of intraflagellar transport (IFT) that traffics proteins required for ciliogenesis, as well as via regulation of ciliary axonemal microtubule stability (Chaya et al., 2014; Craft et al., 2015; Maurya et al., 2019; Phirke et al., 2011; Satoda et al., 2022; Yi et al., 2018). We and others previously showed that in *C. elegans* , the loss of *dyf-18* CCRK and *dyf-5* RCK homologs elongates cilia, while conversely, overexpression of *dyf-5* ( *dyf-5(XS)* ) results in severely truncated cilia (Burghoorn et al., 2007; Maurya et al., 2019). DYF-18-mediated potentiation is required to fully activate DYF-5 function in *C. elegans* similar to observations in mammalian tissues and *Chlamydomonas* (Fu et al., 2005; Maurya et al., 2019; Yang et al., 2013), highlighting the deep functional conservation of this cascade in regulating cilia length.

In addition to CCRK and RCK, the CDKL5 and its *C. elegans* ortholog CDKL-1 kinases have also been implicated in cilia length control (Canning et al., 2018; Hu et al., 2015; Tam et al., 2013). The lengths of the middle (or proximal) segments in a subset of *C. elegans* sensory cilia with simple rod-like structures including those of the ADL sensory neurons are elongated in *cdkl-1* mutants (Park et al., 2021). The middle segments of ADL cilia are also elongated in *dyf-18* but not *dyf-5* mutants, and DYF-18 regulates CDKL-1 ciliary localization in ADL (Park et al., 2021). Similarly, LF2 CCRK regulates the localization of the LF5 CDKL5 ortholog in *Chlamydomonas* flagella (Tam et al., 2013). Together with the observation that ADL cilia are not further elongated in *dyf-18; cdkl-1* double mutants (Park et al., 2021), CDKL-1 has been suggested to act downstream of DYF-18 but independently of DYF-5 to regulate cilia length (Park et al., 2021). However, it remains possible that cilia lengths are at a maximal ceiling in each single mutant precluding accurate interpretation of the double mutant phenotype. Moreover, whether the proposed genetic interactions of CDKL-1 with DYF-18 and DYF-5 are similar in sensory neuron types with different cilia structures is unclear *.*

The severe truncation of the simple rod-like and complex cilia morphologies of the ASH nociceptive and AWA olfactory neurons in *C. elegans* in *dyf-5(XS)* animals (Figure 1A-D) (Burghoorn et al., 2007; Maurya et al., 2019) provides a tool to further dissect the genetic relationships of CDKL-1 and DYF-18 with DYF-5 in these neuron types. While a subset of rod-like cilia containing well-defined ciliary middle segments is markedly elongated in *cdkl-1* mutants (Canning et al., 2018; Park et al., 2021), the overall lengths of the rod-like cilia of the ASH neurons are more weakly affected in this mutant background with a broader distribution of cilia lengths as reported previously (Park et al., 2021) (Figure 1A-B). The morphology of the AWA cilia that lack clearly demarcated middle and distal ciliary segments was also only weakly affected in *cdkl-1* mutants (Figure 1C-D), pointing to a distinct role for this kinase in different cilia types. Loss of *cdkl-1* had no effect on the truncated cilia phenotypes in either ASH or AWA in *dyf-5(XS)* animals (Figure 1A-D), suggesting that similar to observations in ADL (Park et al., 2021), CDKL-1 is also unlikely to act in a linear pathway with DYF-5 to regulate cilia length in ASH or AWA.

While AWA cilia are severely truncated in *dyf-5(XS)* animals, loss of *dyf-18* in this overexpression background is sufficient to restore AWA cilia to wild-type-like morphologies (Figure 1C-D) (Maurya et al., 2019). We previously proposed that DYF-18 is required to fully activate DYF-5 such that in *dyf-18* mutants, overexpression of DYF-5 is sufficient to inhibit AWA cilia elongation and promote ciliary branching but is not sufficient to drive cilia truncation (Maurya et al., 2019). If CDKL-1 acts in parallel to DYF-18 to regulate DYF-5 function, effects of *cdkl-1* loss of function on *dyf-5(XS)* activity may be masked by DYF-18-mediated potentiation of DYF-5. In this case, mutations in both *dyf-18* and *cdkl-1* should result in complete loss of *dyf-5(XS)* function and an elongated cilium phenotype. However, the AWA cilia phenotypes of *dyf-18; cdkl-1; dyf-5(XS)* animals were similar to those of *dyf-18; dyf-5(XS)* animals alone (Figure 1C-D). Similarly, while loss of *dyf-18* restored the truncated ASH cilia lengths to wild-type levels in *dyf-5(XS)* animals, the cilia length phenotype of *dyf-18; cdkl-1; dyf-5(XS)* animals were again similar to those of *dyf-18; dyf-5(XS)* animals (Figure 1A-B). Together, these observations suggest that CDKL-1 likely does not act in parallel with DYF-18 to regulate DYF-5 function and cilia morphology in either ASH or AWA.

Results shown here confirm previous observations that CDKL-1 and DYF-5 act in independent pathways to regulate cilia length in *C. elegans* and extend this conclusion to additional cilia types. Moreover, our findings indicate that while DYF-18 and DYF-5 regulate cilia length in sensory neurons with both simple and complex cilia morphologies, the contribution of CDKL-1 to cilia length control appears to be largely restricted to rod-like sensory cilia (Figure 1E). We suggest that distinct kinase pathways operate via targeting partly overlapping sets of substrates to regulate lengths and morphologies of cilia with unique underlying axonemal ultrastructures.

## Methods

***C. elegans* growth and strain generation**

Worms were grown on *E. coli* OP50 bacteria according to standard procedures. Mutant strains were generated using standard genetic methods and confirmed using PCR-based genotyping. Animals were maintained with plentiful food and uncrowded conditions for at least two generations prior to analyses. The *oyEx691* extrachromosomal array was generated by injecting *sra-6* p:: *myr-gfp* at 10 ng/µl and *unc-122* p:: *gfp* at 50 ng/µl concentration in N2 worms.

**Microscopy**

To image cilia, animals were anesthetized with 10 mM tetramisole hydrochloride (Sigma) and mounted on 2-10% agarose pads in water. A spinning disc confocal microscope (Zeiss Axio Observer with a Yokogawa CSU-22 spinning disk confocal head) was used to image cilia using an 100X objective with 0.25 μm *z* -steps. Maximum intensity projections were generated using SlideBook 6.0 software (3i, Intelligent Imaging Innovations). For visualization, images were false colored and adjusted in ImageJ (NIH) for brightness and contrast.

**Quantification and statistical analyses**

The SPSS 25 statistical analyses package (IBM) was used to perform statistical tests. AWA cilia categories were treated as ordinal variables. One-way ANOVA with Bonferroni post hoc corrections for multiple comparisons were used for ASH cilia length distributions. A non-parametric Kruskal-Wallis test with Bonferroni posthoc corrections for multiple comparisons was used for data with AWA cilia categories that display non-normal distributions.

## Reagents

**Table 1.** List of strains used in this work.

**Table:** 

**Strain**	**Genotype**
PY11513	*oyEx691* [ *sra-6* p:: *myr-gfp* ; *unc-122* p:: *gfp* ]
PY11569	*cdkl-1(ok2694)* ; *oyEx691* [ *sra-6* p:: *myr-gfp* ; *unc-122* p:: *gfp* ]
PY11570	*gjIs831* [ *dyf-5(XS)* ; *elt-2* p:: *gfp* ]; *oyEx691* [ *sra-6* p:: *myr-gfp* ; *unc-122* p:: *gfp* ]
PY11571	*cdkl-1(ok2694)* ; *gjIs831* [ *dyf-5(XS)* ; *elt-2* p:: *gfp* ]; *oyEx691* [ *sra-6* p:: *myr-gfp* ; *unc-122* p:: *gfp* ]
PY11572	*dyf-18(ok200)* ; *gjIs831* [ *dyf-5(XS)* ; *elt-2* p:: *gfp* ]; *oyEx691* [ *sra-6* p:: *myr-gfp* ; *unc-122* p:: *gfp* ]
PY11573	*dyf-18(ok200)* ; *cdkl-1(ok2694)* ; *gjIs831* [ *dyf-5(XS)* ; *elt-2* p:: *gfp* ]; *oyEx691* [ *sra-6* p:: *myr-gfp* ; *unc-122* p:: *gfp* ]
PY11404	*oyIs87* [ *gpa-4Δ6* p:: *myr-gfp* ]
PY11551	*oyIs87* [ *gpa-4Δ6* p:: *myr-gfp* ]; *cdkl-1(ok2694)*
PY11574	*oyIs87* [ *gpa-4Δ6* p:: *myr-gfp* ]; *gjIs831* [ *dyf-5(XS); elt-2* p:: *gfp* ]
PY11575	*cdkl-1(ok2694)* ; *oyIs87* [ *gpa-4Δ6* p:: *myr-gfp* ]; *gjIs831* [ *dyf-5XS; elt-2* p:: *gfp* ]
PY11576	*dyf-18(ok200)* ; *oyIs87* [ *gpa-4Δ6* p:: *myr-gfp* ]; *gjIs831* [ *dyf-5(XS)* ; *elt-2* p:: *gfp* ]
PY11577	*dyf-18(ok200)* ; *cdkl-1(ok2694)* ; *oyIs87* [ *gpa-4Δ6* p:: *myr-gfp* ]; *gjIs831* [ *dyf-5(XS)* ; *elt-2* p:: *gfp* ]
